# Evolution, not transgenerational plasticity, explains the adaptive divergence of acorn ant thermal tolerance across an urban–rural temperature cline

**DOI:** 10.1111/eva.12826

**Published:** 2019-07-18

**Authors:** Ryan A. Martin, Lacy D. Chick, Aaron R. Yilmaz, Sarah E. Diamond

**Affiliations:** ^1^ Department of Biology Case Western Reserve University Cleveland Ohio; ^2^ Present address: The Holden Arboretum Kirtland Ohio

**Keywords:** adaptation, contemporary evolution, global change, maternal effect, speciation, thermal physiology, urban evolution

## Abstract

Although studies increasingly disentangle phenotypic plasticity from evolutionary responses to environmental change, few test for transgenerational plasticity in this context. Here, we evaluate whether phenotypic divergence of acorn ants in response to urbanization is driven by transgenerational plasticity rather than evolution. F2 generation worker ants (offspring of laboratory‐born queens) exhibited similar divergence among urban and rural populations as field‐born worker ants, suggesting that evolutionary divergence rather than transgenerational plasticity was primarily responsible for shifts toward higher heat tolerance and diminished cold tolerance in urban acorn ants. Hybrid offspring from matings between urban and rural populations also indicated that evolutionary divergence was likely the primary mechanism underlying population differences in thermal tolerance. Specifically, thermal tolerance traits were not inherited either maternally or paternally in the hybrid pairings as would be expected for strong parental or grandparental effects mediated through a single sex. Urban–rural hybrid offspring provided further insight into the genetic architecture of thermal adaptation. Heat tolerance of hybrids more resembled the urban–urban pure type, whereas cold tolerance of hybrids more resembled the rural–rural pure type. As a consequence, thermal tolerance traits in this system appear to be influenced by dominance rather than being purely additive traits, and heat and cold tolerance might be determined by separate genes. Though transgenerational plasticity does not appear to explain divergence of acorn ant thermal tolerance, its role in divergence of other traits and across other urbanization gradients merits further study.

## 1 INTRODUCTION

Responses to changing environments can occur either through evolutionary change or through existing phenotypic plasticity (Diamond & Martin, 2016). However, the mechanisms underlying observed phenotypic responses to rapid environmental changes, including through anthropogenic effects, are largely unknown for most organisms, as distinguishing plastic from evolved responses is often challenging (Merilä & Hendry, 2014). Indeed, while the dichotomy between purely plastic and purely evolutionary responses is important and real, the two mechanisms can interact and influence each other (Diamond & Martin, 2016; Ghalambor, McKay, Carroll, & Reznick, 2007; Kelly, 2019). This more complex view posits that along with the concept that plasticity itself evolves (DeWitt & Scheiner, 2004), plastic responses to novel environments may often precede and shape the speed, direction, and magnitude of evolutionary change, thereby facilitating adaptation (i.e., *plasticity‐first hypothesis* or *genetic accommodation*; for theoretical considerations, see Levis & Pfennig, 2016; Price, Qvarnström, & Irwin, 2003; West‐Eberhard, 2003, and for empirical examples, see Corl et al., 2018; Hua et al., 2015; Levis, Isdaner, & Pfennig, 2018). Transgenerational plasticity—the influence of parents' environment on the phenotype of their offspring independent from the effects of genetic inheritance (Bernardo, 1996; Marshall & Uller, 2007; Mousseau & Fox, 1998)—may be especially effective in facilitating adaptive evolution to novel environments, as parents can alter offspring development in anticipation of the environment they will later experience (Badyaev, 2008). Despite this promise, there are few examples of transgenerational plasticity facilitating divergence in response to the altered selective regimes of novel environments (Badyaev, Hill, Beck, et al., 2002; Badyaev, Hill, & Whittingham, 2002; Pfennig & Martin, 2009, 2010).

Cities are an emerging experimental model system for studying plastic and evolutionary responses to rapid anthropogenic change (Rivkin et al., 2019), and a number of studies find evidence that populations are indeed evolving in response to selective pressures associated with urbanization over contemporary timescales (Johnson & Munshi‐South, 2017). For example, research suggests that urban populations of the Puerto Rican crested anole (*Anolis cristatellu*s) have evolved longer limbs and increased toe pad lamellae in response to selection for locomotor performance on artificial surfaces (Winchell, Reynolds, Prado‐Irwin, Puente‐Rolón, & Revell, 2016). This and other studies have also found considerable within‐generational phenotypic plasticity in response to urban environments (Diamond, Chick, Perez, Strickler, & Martin, 2017, 2018; Gorton, Moeller, & Tiffin, 2018). However, evaluating whether transgenerational plasticity contributes to divergence in urban habitats has not yet been explored.

A classic approach for disentangling plastic responses occurring both within and across generations from evolved responses is to compare field phenotypic measures between populations with those from individuals of the same populations bred and reared in a controlled, shared environment (i.e., a common‐garden experiment) across multiple generations (Donihue & Lambert, 2015; Merilä & Hendry, 2014). By controlling for environmental differences across populations in the common garden, researchers can isolate the genetic component, if any, of the phenotypic divergence measured in the field. Although this approach is conceptually simple, such experiments are often labor‐intensive and time‐consuming and might be challenging or impossible for species that are difficult to rear and breed in the laboratory (Donelson, Salinas, Munday, & Shama, 2018). Consequently, most urban evolution studies to date have only reared field‐caught juveniles or a single generation in the laboratory and as a result cannot fully disentangle transgenerational phenotypic plasticity from within‐generation plasticity and purely genetic divergence (but see Brans et al., 2017).

Here, we use a laboratory common‐garden experiment with a multi‐generational breeding design to test for transgenerational plasticity as an explanation for divergence between urban and rural populations of the acorn ant (*Temnothorax curvispinosus*, Mayr 1866). We focus on thermal tolerance traits (heat and cold tolerance) and their response to urban heat island effects along the urbanization gradient of Cleveland, Ohio, USA. We have previously found that field‐born workers and F1 offspring (laboratory‐born workers of field‐caught queens) of urban acorn ant populations reared under common garden exhibit increased heat tolerance and diminished cold tolerance compared with rural populations; this pattern of divergence among urban and rural acorn ant populations is apparent for geographically isolated cities, including Cleveland, Ohio and Knoxville, Tennessee (Diamond et al., 2017, 2018). Moreover, in both cities we found that urban colonies produced more alates (i.e., reproductive offspring) under typical urban rearing temperatures, and rural colonies produced more alates when reared at typical rural rearing temperatures, resulting in fitness trade‐offs across environments for each population (Diamond et al., 2018). These results strongly suggest that *T. curvispinosus* has adaptively evolved to the warmer environmental temperatures of the urban environment (i.e., the urban heat island effect). However, our design could not detect or rule out transgenerational plasticity as an alternative mechanism behind this adaptive divergence.

In this study, we tested the heat and cold tolerance of F2 offspring, laboratory‐born workers of laboratory‐born queens. We tested the offspring of pure‐type rural population matings, pure‐type urban population matings, and hybrids, both where the female was from the rural population and the male from the urban population and vice versa. If transgenerational plasticity were responsible for the increased heat tolerance and diminished cold tolerance we documented previously in urban population acorn ants, then we would expect these phenotypic differences to disappear or weaken in the F2 offspring comparisons of pure‐type urban and rural populations. If transgenerational plasticity plays only a minor to no role, we would expect the increased heat tolerance and diminished cold tolerance of pure‐type urban populations relative to rural populations to remain in the F2 offspring. The hybrid matings further evaluate the role of transgenerational plasticity, where parental (or grandparental) effects mediated through a single sex should differentially affect hybrid offspring depending on the direction of the urban–rural mating. Additionally, comparisons among and against the hybrid tolerance phenotypes allowed us to gain further insight into the genetic architecture underlying heat and cold tolerance. For example, these comparisons allowed us to distinguish a purely additive genetic model of trait inheritance from alternatives, such as dominance or transgressive segregation.

## 2 MATERIALS AND METHODS

### 2.1 Colony collections

We collected queenright (queen present) acorn ant (*T. curvispinosus*) colonies in early summer 2017 (June 6, 2017–July 21, 2017) from urban and rural sites across Cleveland, Ohio (42°N latitude). To identify urban and rural sites, we used percent developed impervious surface area (ISA) (Imhoff, Zhang, Wolfe, & Bounoua, 2010), such that urban sites were categorized as 40%–50% ISA, and rural sites were categorized as 0% ISA (see Table S1 for site details). Our previous research showed that these ISA designations for urban versus rural sites were sufficient to generate a temperature difference (at the microhabitat level of individual acorns) in excess of 4°C in Cleveland, Ohio (Diamond et al., 2018).

### 2.2 Mating design and common‐garden rearing

We returned field‐caught colonies to the laboratory and placed them under conditions to facilitate the production of male and female alates (or winged, sexual individuals), including a warm, diurnally fluctuating thermal regime (30°C daytime and 26°C nighttime temperature) synced with a 14:10 short‐day photoperiod (following Stuart, Gresham‐Bissett, & Alloway, 1993; Percival 36‐VL growth chambers). Each colony was maintained individually in a 120‐ml plastic cup. Resource tubes with sugar water (25% solution) and plain tap water were provided to colonies along with a continuous supply of dead mealworms. Once alates were produced within the colonies, we paired males and females in four mating crosses, including two pure‐type matings, rural–rural and urban–urban, and two hybrid matings, rural–urban (with the maternal source population listed first) and urban–rural. Because acorn ants are suggested to have male‐biased dispersal (Stuart et al., 1993), we provided several female alates (typically five alates) from the same colony with males from several different colonies (typically 10 alates, all of which were distinct from the female alate source colony). We enclosed alates from each cross in separate glass aquaria (38 L capacity) with a mesh netting top. Alates were provided with the same resources as their source colonies, including water and sugar tubes plus dead mealworms. We provided alates with several uninhabited, hollowed‐out acorns that we had sliced horizontally and held together with garden twine. This allowed us to check on the mating status of the paired alates, specifically whether the ants had shed their wings (an indication that they have mated, Herbers, 1990) and whether mated female alates began to lay eggs. All mated females began new egg production by December 7, 2017.

Once mated female alates began to produce eggs, we placed these newly established colonies individually within 120‐ml plastic cups and provided them with water and sugar resource tubes and dead mealworms. We held these colonies at a constant 25°C (on a 14:10 photoperiod), as this is the optimal temperature for brood development (Diamond et al., 2013). After the newly laid eggs developed into workers, we tested the thermal tolerance of these workers (i.e., the second generation of acorn ant workers reared entirely within the laboratory environment).

### 2.3 Thermal tolerance

We used a dynamic temperature ramping protocol to assess the critical thermal maximum and minimum (CT_max_ and CT_min_), each defined as the loss of muscular coordination, which yield ecologically relevant limits on performance and serve as our measures of heat and cold tolerance (Terblanche et al., 2011). Although the frequency at which acorn ants approach and exceed their thermal tolerances in nature is not known, importantly, thermal tolerance traits are correlated with sub‐lethal performance in *T. curvispinosus*. Specifically, the response of CT_max_ to rearing temperature is significantly and positively associated with the response of development rate to rearing temperature (Penick, Diamond, Sanders, & Dunn, 2017). We tested workers individually for either CT_max_ or CT_min_, as the assessment of thermal tolerance is a semi‐destructive process that precludes assessment of both CT_max_ and CT_min_ on the same individual. Ants were placed individually into 1.5‐ml Eppendorf tubes with a cotton plug in the lid. Temperatures were manipulated using a dry‐block incubator (Boekel Scientific TropiCooler) and increased or decreased at a rate of 1°C/min. Initial temperature was 34°C for the estimation of CT_max_ and was 16°C for CT_min_. These starting temperatures lie outside the range needed to induce loss of muscular coordination or death.

Thermal tolerances were assessed between August 30, 2018, and September 2, 2018. We tested a total of 487 individuals for thermal tolerance, 251 CT_max_ and 236 CT_min_ from 18 different colonies. For the pure mating types, we had four urban–urban colonies and four rural–rural colonies. For the hybrid mating types, we had five urban–rural (with the origin of the maternal population listed first) colonies and five rural–urban colonies. The number of individuals tested per colony (and within each tolerance type, heat or cold tolerance) ranged from 5 to 24 with a mean and *SD* of 13.6 and 5.17 (see also Table S2).

### 2.4 Field‐caught thermal tolerance

Although not part of our original design, we also collected data on field‐caught acorn ant thermal tolerances. These colonies, including nine rural colonies and 15 urban colonies, were collected contemporaneously (same time interval and field sites) with those colonies that served as the parental generation in the transgenerational plasticity experiment. Colonies from urban and rural populations were acclimated for 1 week at a constant 25°C (14:10 photoperiod) prior to the assessment of thermal tolerance. Five individuals per colony were tested for CT_max_, and another five were tested for CT_min_ using the same protocols as in the transgenerational plasticity experiment. These data allowed us to compare the magnitude of field‐caught urban–rural population divergence in heat and cold tolerance with that of the F2 urban–rural (pure type) population divergence. If the magnitude of divergence is similar, and specifically if it does not decline between the field‐caught and F2 generations, this would suggest little to no role for transgenerational plasticity in explaining the evolutionary divergence in tolerance. Ideally, we would also explore the magnitude of divergence across field‐caught, F1, and F2 workers from the same colony, but this was not possible owing to needing the colonies to be of large enough size to generate reproductive ants (alates). F1 workers from other experiments show evolutionary divergence in acorn ant heat and cold tolerance across urban and rural populations (Diamond et al., 2017, 2018), but the methods used in these experiments were not comparable to the methods used here. Because these differences in methods, that is, different rates of temperature change during the thermal tolerance assay or different thermal fluctuations during the laboratory acclimation period, yield plastic effects on heat and cold tolerance on the same order of magnitude as the evolutionary divergence, we could not directly compare these estimates with the F2 ants in our current study.

### 2.5 Statistical analyses

#### 2.5.1 F2 ants

We fit linear mixed‐effects models with either CT_max_ or CT_min_ as the response variable and the type of mating (the two pure types, urban–urban and rural–rural, and the two hybrid types with the population origin of the mother listed first, urban–rural and rural–urban) as a predictor variable (treated as a categorical variable with four levels). We used the *lme* function from the *nlme* library in R (Pinheiro, Bates, DebRoy, & Sarkar, 2018; R Core & Team, 2018) to perform these models. Colony identity was included as a random intercept in each model to account for autocorrelation among individuals from the same colony. The statistical significance of mating type was assessed using likelihood ratio tests. Post hoc analyses to assess the significant differences between factor levels of the type of mating predictor variable were performed using the *emmeans* function (from the eponymous library) with all pairwise comparisons (Lenth, 2018).

To model tolerance breadth (the difference between colony mean CT_max_ and colony mean CT_min_), we fit a generalized linear model with tolerance breadth as the response and type of mating as a predictor. We modeled tolerance breadth using a Gaussian probability distribution and log link function owing to skew in the untransformed data. Because hybrid tolerance responses were indistinguishable from one another and because we were more limited in statistical power after computing colony‐level means for heat and cold tolerance, we fit a subsequent model wherein each of the pure types (urban–urban and rural–rural) was compared with the hybrid types (considered as a single group). We performed the post hoc analysis on this model. Although a strict test of maternal effects would involve comparisons of the urban maternal pairings versus rural pairings, our initial analyses showed little evidence of maternal effects (see below), so we used the particular analyses for thermal tolerance breadth to explore the genetic architecture underlying heat versus cold tolerance and hence justified our combining of offspring results for the hybrid matings.

#### 2.5.2 Field‐caught ants

To explore whether field‐caught ants exhibited a similar magnitude of urban–rural divergence in thermal tolerance traits as F2 ants, we fit linear mixed‐effects models with thermal tolerance (either CT_max_ or CT_min_) as the response, and population (urban vs. rural), generation (field‐caught vs. F2), and their interaction as categorical predictors. We included colony identity as a random intercept to account for autocorrelation among individuals from the same colony.

## 3 RESULTS

### 3.1 F2 ants

**Figure 1:**
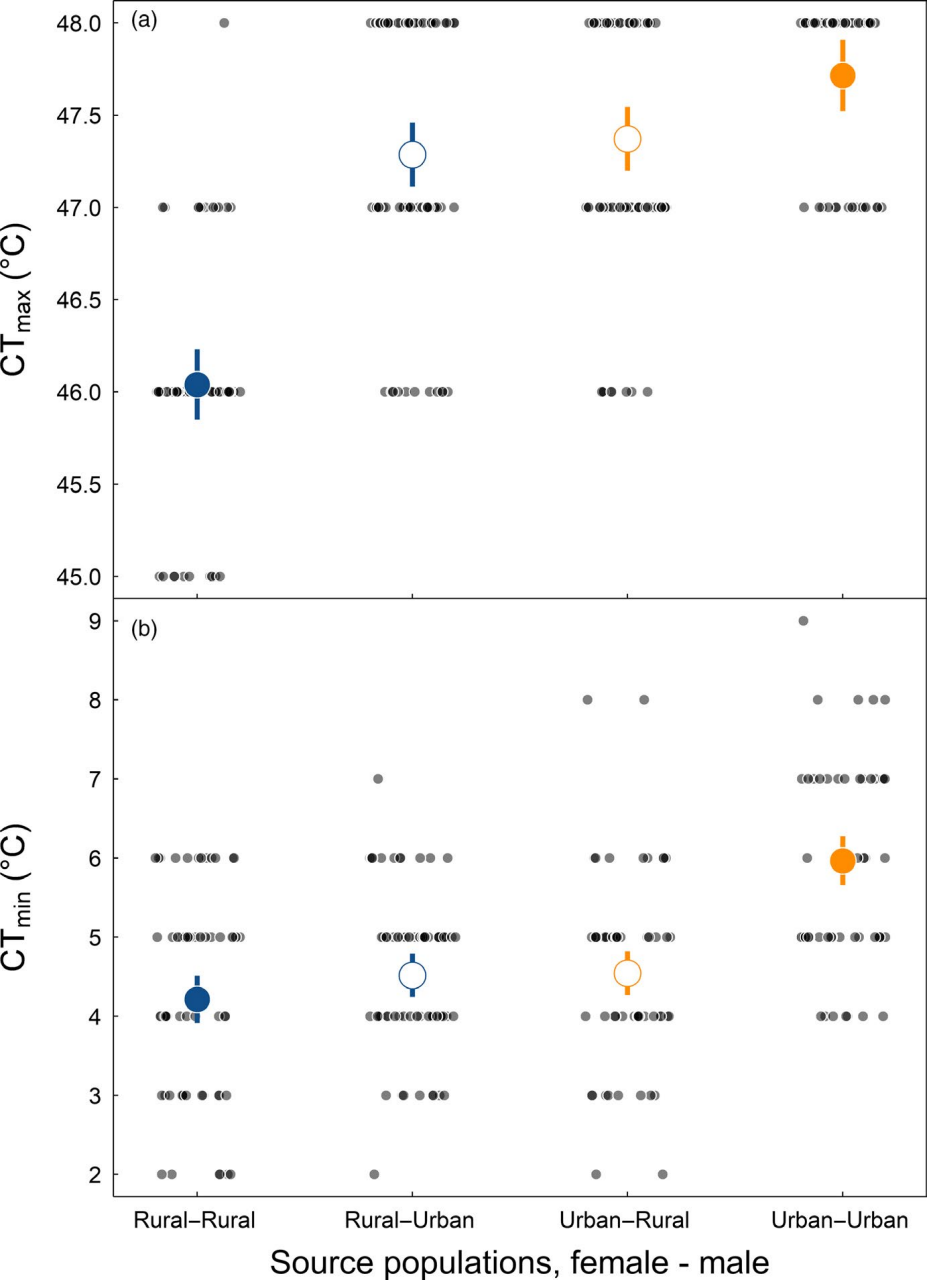
**(**a) Heat tolerance (CT_max_, °C) and (b) cold tolerance (CT_min_, °C) as a function of pure‐type and hybrid matings across urban and rural acorn ant populations. Small points indicate tolerance values of individual worker ants from each mating type (with the maternal source population listed first): rural–rural, rural–urban, urban–rural, and urban–urban. Predicted mean heat tolerances, cold tolerances, and standard errors from linear mixed‐effects models that account for colony‐level autocorrelation are presented in large points (means) and line segments (standard errors). Pure types are in filled symbols (blue for rural and orange for urban). Open symbols represent hybrid matings, with the color reflecting the maternal population origin

We found no evidence that the divergence in heat and cold tolerance among urban and rural acorn ant populations was a result of transgenerational plasticity. Likelihood ratio tests for the effect of mating type on heat tolerance and cold tolerance were statistically significant (CT_max_: *χ* ^2^ = 44.6; *p* = 1.12E−09; *df* = 3; CT_min_: *χ* ^2^ = 20; *p* = 0.00017; *df* = 3). F2 offspring of male and female reproductive ants born, reared, and mated within the laboratory environment exhibited significantly elevated heat tolerance and significantly diminished cold tolerance in urban–urban crosses as compared with rural–rural crosses, as revealed by post hoc tests (Table 1, Figure 1).

**Table 1:** Post hoc analyses of the mating type term for models of CT_max_, CT_min_, and tolerance breadth are provided, including estimates of the contrast, their standard errors, test statistics, and *p*‐values for the pairwise differences between factor levels of the mating type term

Tolerance type	Contrast	Estimate	*SE*	Test statistic	*p*
CT_max_	rur‐rur ‐ rur‐urb	−1.25	0.257	−4.84	**0.00132**
	rur‐rur ‐ urb‐rur	−1.33	0.257	−5.17	**0.000723**
	rur‐rur ‐ urb‐urb	−1.67	0.272	−6.17	**0.000128**
	rur‐urb ‐ urb‐rur	−0.0855	0.245	−0.35	0.985
	rur‐urb ‐ urb‐urb	−0.428	0.259	−1.65	0.384
	urb‐rur ‐ urb‐urb	−0.343	0.26	−1.32	0.566
CT_min_	rur‐rur ‐ rur‐urb	−0.304	0.405	−0.75	0.875
	rur‐rur ‐ urb‐rur	−0.331	0.408	−0.813	0.848
	rur‐rur ‐ urb‐urb	−1.75	0.43	−4.08	**0.00548**
	rur‐urb ‐ urb‐rur	−0.0276	0.39	−0.0709	1
	rur‐urb ‐ urb‐urb	−1.45	0.412	−3.51	**0.0161**
	urb‐rur ‐ urb‐urb	−1.42	0.415	−3.42	**0.019**
Tolerance breadth	hybrid ‐ rur‐rur	0.0231	0.00984	2.35	**0.0490**
	hybrid ‐ urb‐urb	0.0260	0.00986	2.64	**0.0225**
	rur‐rur ‐ urb‐urb	0.00290	0.0119	0.244	0.968

Note

Hybrid factor levels are combined for the analysis of tolerance breadth and that estimates and test statistics are reported on the natural log scale. Statistically significant *p‐*values at the 0.05 level are indicated in bold font. All values are presented to three significant digits.

The results of the hybrid pairings (female–male), urban–rural and rural–urban, revealed further insights into the contemporary evolution of thermal tolerance traits in response to urbanization. First, the fact that the hybrid matings were successful in producing offspring indicates no evidence of behavioral prezygotic or intrinsic postzygotic reproductive isolation among urban and rural populations. It is possible that F2 offspring may exhibit later signs of reproductive isolation (hybrid inviability or sterility). We also note that male F2 hybrid incompatibility is unclear from our experimental design and study system as males are haploid clones of diploid queens, and thus, another round of mating (F3) would be required to test this possibility (Koevoets & Beukeboom, 2009). In any case, these are secondary goals of our study.

Interestingly, hybrids were more similar to either the rural–rural or urban–urban pure types depending on whether heat tolerance or cold tolerance was assessed. The heat tolerance of hybrids more resembled the urban–urban pure type than the rural–rural pure type (Figure 1a), whereas the cold tolerance of hybrids more resembled the rural–rural pure type than the urban–urban pure type (Figure 1b). This provides further evidence that divergence in thermal tolerance does not stem from transgenerational plasticity, as the offspring did not consistently resemble a specific parental or grandparental population as would be expected if transgenerational plasticity was mediated through a single sex. Moreover, this differential matching of hybrids and pure types across heat and cold tolerance potentially suggests some degree of genetic decoupling of the traits.

**Figure 2:**
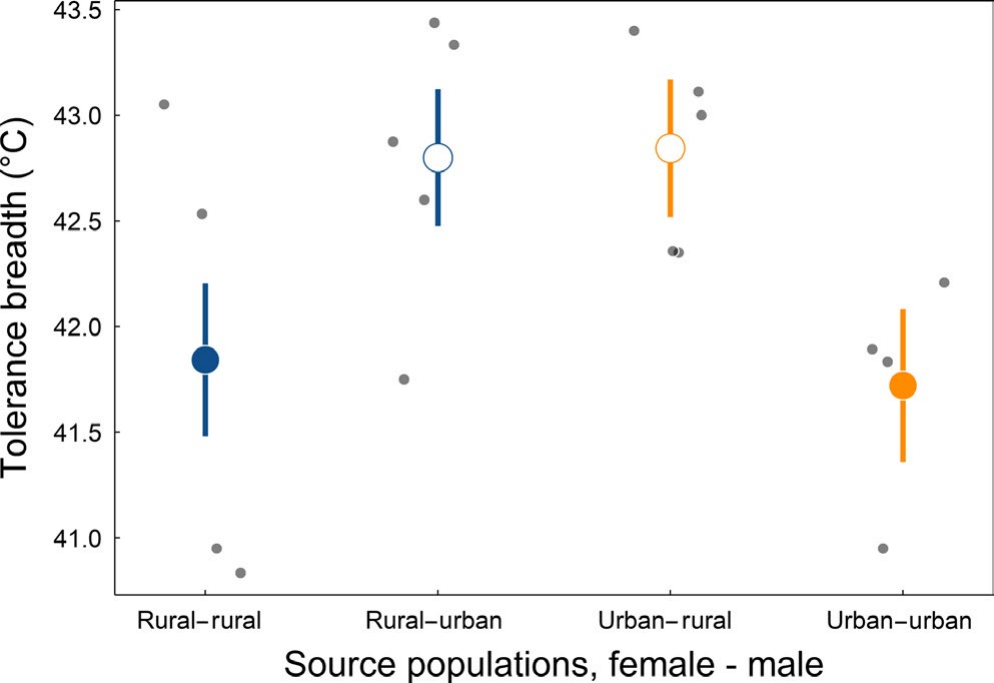
Tolerance breadth (°C) as a function of pure‐type and hybrid matings across urban and rural acorn ant populations. Small points indicate tolerance breadth values of whole colonies (colony mean CT_max_ ‐ colony mean CT_min_) from each mating type (with the maternal source population listed first): rural–rural, rural–urban, urban–rural, and urban–urban. Predicted mean tolerance breadths and standard errors from a generalized linear model are presented in large points (means) and line segments (standard errors). Pure types are in filled symbols (blue for rural and orange for urban). Open symbols represent hybrid matings, with the color reflecting the maternal population origin

We further explored the potential ecological consequences of the differential matching of hybrid and pure‐type heat and cold tolerance phenotypes by computing their tolerance breadth (difference between colony mean CT_max_ and CT_min_). The likelihood ratio test for the effect of mating type on tolerance breadth was statistically significant (*χ* ^2^ = 9.22; *p* = 0.0266; *df* = 2). Post hoc tests revealed that hybrids exhibited greater tolerance breadth than either of the pure types since the hybrids possessed both the superior heat tolerance value (high CT_max_) of urban populations and the superior cold tolerance value (low CT_min_) of rural populations (Figure 2; Table 1).

### 3.2 Field‐caught ants

We found a similar magnitude of urban–rural divergence in thermal tolerance traits across field‐caught and F2 generations. Likelihood ratio tests for the effects of population, generation, and their interaction on heat tolerance revealed a significant effect of population, but not of generation or the interaction of generation with population (population: *χ* ^2^ = 41.1; *p* = 1.43E−10; *df* = 1; generation: *χ* ^2^ = 0.261; *p* = 0.609; *df* = 1; population × generation: *χ* ^2^ = 1.70; *p* = 0.193; *df* = 1). We found a similar pattern for cold tolerance. Likelihood ratio tests for the effects of population, generation, and their interaction on cold tolerance revealed a significant effect of population, but not of generation or the interaction of generation with population (population: *χ* ^2^ = 16.7; *p* = 4.50E−05; *df* = 1; generation: *χ* ^2^ = 3.80; *p* = 0.0514; *df* = 1; population × generation: *χ* ^2^ = 3.44; *p* = 0.0635; *df* = 1).

## 4 DISCUSSION

Populations can adaptively respond to rapidly changing environments through evolutionary change and phenotypic plasticity occurring within or across generations or through their interaction (Merilä & Hendry, 2014). Although distinguishing among these mechanisms is often difficult, doing so is necessary to understand and accurately predict future responses to rapid environmental change (Urban et al., 2016). Here, we used a multi‐generational common‐garden study to discriminate between the effects of transgenerational plasticity and the effects of evolutionary change. We explored this question in context of the adaptive divergence of thermal tolerance traits between urban and rural populations of the acorn ant *T. curvispinosus*. We found no evidence that transgenerational plasticity, rather than evolution, was the primary mechanism of divergence between urban and rural populations in thermal tolerance.

**Figure 3:**
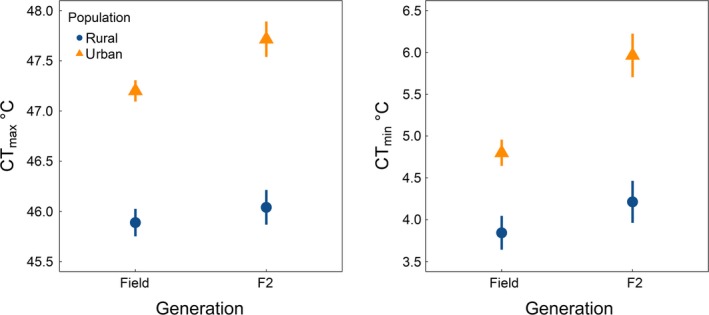
Comparison of field‐caught and common‐garden reared F2 acorn ant worker heat and cold tolerances (CT_max _and CT_min_, °C). Predicted mean heat tolerance and cold tolerance are shown with their standard errors for each combination of population (urban and rural) and generation (field‐caught and F2) from linear mixed‐effects models that account for colony‐level autocorrelation

Transgenerational plasticity can persist across multiple generations (reviewed in Bernardo, 1996; see also Shama & Wegner, 2014), and our design could not entirely rule out transgenerational environmental effects persisting across grandparental or more distant generations. However, such effects appear unlikely for two reasons. First, the magnitude and direction of shifts toward better heat tolerance and worse cold tolerance in urban populations were maintained between field‐collected and F2 worker ants after two generations of rearing in a common‐garden laboratory environment (Figure 3). Moreover, we found that the thermal tolerance traits were not inherited either maternally or paternally in the hybrid pairings as would be expected for strong parental or grandparental effects mediated through a single sex (Figure 1, Table 1). Ideally, one would perform manipulative experiments starting from the same genetic material over multiple generations beyond the F2 generation.

Our results align with those of the few other studies that have used multi‐generation common‐garden experiments in the context of thermal tolerance divergence to urban heat islands (Brans et al., 2017). Yet, the overall importance of transgenerational plasticity for evolutionary responses to novel environments is far from known. The thermal environment experienced by parents does appear to adaptively affect the thermal tolerance of their offspring in other contexts and systems, providing strong examples of anticipatory parental effects (Chirgwin, Marshall, Sgrò, & Monro, 2018; Massamba‐N'Siala, Prevedelli, & Simonini, 2014). However, a recent comprehensive synthesis of transgenerational plasticity across taxa and traits found relatively weak effects overall (Uller, Nakagawa, & English, 2013). Adaptive transgenerational plasticity in thermal tolerance could therefore prove to be relatively uncommon, albeit with important exceptions, such as those mentioned above. Alternatively, previous studies might not have been designed to detect such transgenerational plasticity effectively or might not have focused on systems where such effects are expected to evolve (Burgess & Marshall, 2014). This may occur if researchers are not actually manipulating the aspects of the environment that are most predictive of the conditions offspring will encounter (Burgess & Marshall, 2014; Uller, 2008).

Is there reason to expect adaptive parental effects to evolve in acorn ants as a response to the urban heat islands? Perhaps not. Acorn ant dispersal ability is quite limited (Herbers & Tucker, 1986; Prebus, 2017), and it is highly unlikely that acorn ants experience a change from urban to rural temperature environments from one generation to the next. Further, within an acorn ant generation, workers experience substantial thermal variation over very rapid spatio‐temporal scales, including diurnally within the acorn ant nest environment and spatially as foragers move throughout the landscape. This thermal variability is greater within urban forest patches, and urban population ants have evolved greater within‐generation thermal plasticity to rapid temperature increases (Diamond, Chick, Perez, Strickler, & Zhao, 2018). Consequently, while both temperature means and variances differ between urban and rural populations, the temporal and spatial scale of this variation is unlikely to select for adaptive transgenerational plasticity.

Although the primary goal of our experiment was to evaluate transgenerational plasticity as an alternative explanation for the adaptive divergence in thermal tolerance between urban and rural *T. curvispinosus* populations, our study is not poised to address the overall presence or absence of environmental parental effects. That is, we cannot rule out *any* role for transgenerational thermal plasticity in this system or others, only that it does not appear to explain the urban–rural divergence in thermal tolerance here (Figures 1, 3; Table 1). Indeed, because we reared acorn ant colonies under nonstressful temperatures within our multi‐generation common‐garden experiment, it remains unclear whether other manipulations of the thermal environment, such as heat stress (Chirgwin et al., 2018) or seasonal temperature variation (Walsh, Whittington, & Funkhouser, 2014), could induce transgenerational effects and whether such effects could have played an initial role in facilitating divergence (Badyaev, Hill, Beck, et al., 2002; Badyaev, Hill, & Whittingham, 2002; Pfennig & Martin, 2009, 2010).

While we did not find evidence for transgenerational plasticity as an explanation of the divergence in heat and cold tolerance traits among urban and rural acorn ant populations, the results of the urban–rural hybrid crosses yielded some surprising insights into the genetic architecture of acorn ant thermal tolerance. Specifically, our comparisons of heat and cold tolerance traits between the pure‐type and hybrid urban–rural crosses revealed that dominant and recessive alleles appear to underlie heat and cold tolerance traits, rather than being determined as purely additive traits. We found that both hybrid crosses resemble urban populations in heat tolerance and achieve greater cold tolerances similar to that of rural populations, rather than exhibiting intermediate phenotypes as would be expected for additive traits (Figure 1, Table 1). Why might both hybrid crosses express the greater heat tolerance of urban populations *and* the greater cold tolerance of rural populations? One explanation is that heat tolerance and cold tolerance are largely determined by different genes, with urban populations contributing a dominant allele(s) for heat tolerance and rural populations a dominant allele(s) for cold tolerance (Chown & Nicolson, 2004). Alternatively, heat and cold tolerance could share genes with alternative conditional mutations (Griffiths et al., 2005), resulting in environmentally dependent dominance. For example, imagine that urban and rural populations are fixed for different alleles of a heat‐shock protein. At low temperatures, the urban allele suffers loss of function and conversely the rural allele loses function at high temperatures, resulting in temperature‐sensitive dominance. The latter scenario would suggest that the losses of cold tolerance in urban population could, in part, be a correlated response to selection for increased heat tolerance in urban habitats. However, heat and cold tolerance are not strongly genetically correlated within colonies (Diamond et al., 2018), suggesting that they may indeed be determined by separate genes. This would also correspond to the general findings from *Drosophila* and other ectotherm systems which show that cold and heat tolerance traits are under independent or semi‐independent genetic control (Hoffmann, Sørensen, & Loeschcke, 2003).

Our research provides a case study in how an urban–rural cline in temperature can be used to explore the mechanisms underlying shifts in thermal tolerance trait values, specifically disentangling evolutionary change from transgenerational plasticity, and to understand the genetic architecture underlying these traits. But what else can we gain from such comparisons? With the growing consensus that evolution can occur in the wild on the scale of human lifetimes (Reznick, Losos, & Travis, 2018), cities might also allow us to peer into the early stages of speciation as differences accumulate among urban and rural populations (Thompson, Rieseberg, & Schluter, 2018). Indeed, as acorn ant populations have evolved divergent thermal tolerances in response to urban heat islands, could this ecologically divergent selection promote speciation as well? Ecologically divergent environments can promote reproductive isolation in several ways. For example, populations may become reproductively isolated by shifting the timing of reproduction between them (Rundle & Nosil, 2005). In the acorn ant system, the date of peak alate production is shifted earlier by as much as 30 days in urban populations compared with rural populations, likely due to the differences in the timing of seasonal temperature cues in rural and urban habitats (Chick, Strickler, Perez, Martin, & Diamond, 2019). This phenological shift in reproduction is thus likely to reduce possibilities for mating between urban and rural populations. In contrast, our laboratory breeding experiment shows that, given the opportunity, urban and rural alates (i.e., reproductive‐caste offspring) will mate and produce viable offspring, revealing a lack of both behavioral prezygotic and intrinsic genetic postzygotic isolating mechanisms. Although, it remains to be determined if hybrids are fertile, and because male alates are haploid, any male sterility corresponding with Haldane's rule would only be expressed by F3 male hybrids who, through recombination, carry alleles from both urban and rural populations (Koevoets & Beukeboom, 2009).

Ecologically divergent environments can also impose reproductive isolation by selecting against migrants and/or hybrid offspring (Nosil, 2004; Nosil, Crespi, & Sandoval, 2003). Local adaptation and divergent selection between urban and rural thermal environments suggest that acorn ant migrants would face negative selection pressures in their non‐native habitat. Hybrid acorn ant offspring, in comparison, inherit the combined thermal tolerance of both their parents, with heat and cold tolerances equaling those of urban and rural populations, respectively (Figure 1, Table 1). This expanded thermal performance (Figure 2, Table 1) breaks the expectation of intermediate hybrid fitness for ecological speciation (Rundle & Nosil, 2005) and suggests that hybridization could lead to population collapse rather than population divergence. We then expect that limited dispersal ability, shifted phenologies, or strong selection against migrants has enabled the evolutionary divergence between urban and rural populations in light of the expanded thermal breadth of their hybrid offspring. However, the evidence for any ongoing speciation between urban and rural population is both preliminary and mixed at this time.

In summary, the results from a two‐generation common‐garden rearing experiment support the conclusion that acorn ants have evolved divergent heat and cold thermal tolerances in response to the selective agent of the urban heat island in Cleveland, Ohio. Surprisingly, thermal tolerance appears to be influenced by dominance rather than being a purely additive trait, and heat and cold tolerance might be determined by separate genes. While urban and rural populations have also diverged in their reproductive phenology, there is no evidence for other pre‐ or postzygotic reproductive isolation between them. *Temnothorax curvispinosus* populations have also diverged in thermal tolerance along at least one other urbanization gradient (Knoxville, Tennessee), and it is an open question remaining to be explored whether the genetic architecture, mating behavior, or hybrid success is convergent across this independent selective gradient. As urban environments provide replicated theaters against which the evolutionary play unfolds, future work aimed at comparisons of the mechanisms of trait evolution across multiple urban heat islands could be especially fruitful.

## CONFLICT OF INTEREST

None declared.

## AUTHOR CONTRIBUTIONS

R.A.M. and S.E.D. designed the study, analyzed the data, and wrote the first draft of the manuscript. All authors collected the data and contributed to revisions.

## Data Availability

Data deposited and available in Dryad: https://doi.org/10.5061/dryad.0r75421.
