# Supplementary material for: Evolution, not transgenerational plasticity, explains the adaptive divergence of acorn ant thermal tolerance across an urban–rural temperature cline
Source: Evol Appl. 2019 Jul 18;12(8):1678–87. doi: 10.1111/eva.12826 (PMC6708418; doi:10.1111/eva.12826)
Supplement: Supplementary file 1 [file EVA-12-1678-s001.docx]

**Supporting Information**

**Table S1**. Geographic coordinates and percent developed impervious surface area (ISA) values (0% ISA indicates rural sites, 40-50% ISA indicates urban sites) of acorn ant collection sites.

| Site name | Longitude | Latitude | Source environment | ISA |
| --- | --- | --- | --- | --- |
| University Farm | -81.4245 | 41.49842 | Rural | 0 |
| Holden Arboretum | -81.3127 | 41.6088 | Rural | 0 |
| Ambler Park | -81.6055 | 41.4976 | Urban | 40 |
| Case Western Reserve University | -81.6137 | 41.50897 | Urban | 49 |
| Forest Hills | -81.5737 | 41.52751 | Urban | 44 |

**Table S2**. Number of individual worker ants (F2 generation) tested for CT_min_ and CT_max_ per each colony. The type of cross (the two hybrids and two pure types) is also indicated with the maternal source population listed first.

| Cross | Number of individuals tested for CT_max_ | Number of individuals tested for CT_min_ |
| --- | --- | --- |
| rur-rur | 15 | 15 |
| rur-rur | 12 | 12 |
| rur-rur | 13 | 12 |
| rur-rur | 20 | 20 |
| rur-urb | 16 | 16 |
| rur-urb | 16 | 16 |
| rur-urb | 24 | 24 |
| rur-urb | 6 | 5 |
| rur-urb | 10 | 10 |
| urb-rur | 20 | 10 |
| urb-rur | 11 | 11 |
| urb-rur | 10 | 10 |
| urb-rur | 14 | 14 |
| urb-rur | 9 | 9 |
| urb-urb | 11 | 9 |
| urb-urb | 8 | 7 |
| urb-urb | 24 | 24 |
| urb-urb | 12 | 12 |
